# Co-inhibition of HDAC and MLL-menin interaction targets MLL-rearranged acute myeloid leukemia cells via disruption of DNA damage checkpoint and DNA repair

**DOI:** 10.1186/s13148-019-0723-0

**Published:** 2019-10-07

**Authors:** Jing Ye, Jie Zha, Yuanfei Shi, Yin Li, Delin Yuan, Qinwei Chen, Fusheng Lin, Zhihong Fang, Yong Yu, Yun Dai, Bing Xu

**Affiliations:** 1 Department of Hematology, The First Affiliated Hospital of Xiamen University and Institute of Hematology, School of Medicine, Xiamen University, Xiamen, China; 2 Department of Oncology, The First Affiliated Hospital of Jinan University, Jinan University, Guangzhou, China; 3 Clinical and Translational Research Center of Shanghai First Maternity and Infant Hospital, School of Life Sciences and Technology, Tongji University, Shanghai, China; 4 Laboratory of Cancer Precision Medicine, The First Hospital of Jilin University, Changchun, China

**Keywords:** MLL-rearrangement, Menin-MLL interaction inhibitor, HDAC inhibitor, DNA damage response, AML

## Abstract

While the aberrant translocation of the mixed-lineage leukemia (MLL) gene drives pathogenesis of acute myeloid leukemia (AML), it represents an independent predictor for poor prognosis of adult AML patients. Thus, small molecule inhibitors targeting menin-MLL fusion protein interaction have been emerging for the treatment of MLL-rearranged AML. As both inhibitors of histone deacetylase (HDAC) and menin-MLL interaction target the transcription-regulatory machinery involving epigenetic regulation of chromatin remodeling that governs the expression of genes involved in tumorigenesis, we hypothesized that these two classes of agents might interact to kill MLL-rearranged (MLL-r) AML cells. Here, we report that the combination treatment with subtoxic doses of the HDAC inhibitor chidamide and the menin-MLL interaction inhibitor MI-3 displayed a highly synergistic anti-tumor activity against human MLL-r AML cells in vitro and in vivo, but not those without this genetic aberration. Mechanistically, co-exposure to chidamide and MI-3 led to robust apoptosis in MLL-r AML cells, in association with loss of mitochondrial membrane potential and a sharp increase in ROS generation. Combined treatment also disrupted DNA damage checkpoint at the level of CHK1 and CHK2 kinases, rather than their upstream kinases (ATR and ATM), as well as DNA repair likely via homologous recombination (HR), but not non-homologous end joining (NHEJ). Genome-wide RNAseq revealed gene expression alterations involving several potential signaling pathways (e.g., cell cycle, DNA repair, MAPK, NF-κB) that might account for or contribute to the mechanisms of action underlying anti-leukemia activity of chidamide and MI-3 as a single agent and particularly in combination in MLL-r AML. Collectively, these findings provide a preclinical basis for further clinical investigation of this novel targeted strategy combining HDAC and Menin-MLL interaction inhibitors to improve therapeutic outcomes in a subset of patients with poor-prognostic MLL-r leukemia.

**Electronic supplementary material:**

The online version of this article (10.1186/s13148-019-0723-0) contains supplementary material, which is available to authorized users.

## Introduction

Cytogenetic abnormalities are closely associated with clinical features and therapeutic responses in acute myeloid leukemia (AML) [1]. Chromosome 11q23 translocations occur in 10% of adult AML patients, while being even more frequent in pediatric cases (35%) [2–4]. In AML, most of 11q23 translocations led to fusion proteins involving the mixed lineage leukemia (MLL) gene that encodes histone lysine methyltransferase 2A (KMT2A) [5], of which more than 70 translocation partners of MLL have been characterized so far [6, 7]. The clinical outcome of patients carrying MLL-rearrangement who often suffer from either failure of induction therapy or disease relapse remains extremely poor, while the response rate reported in adult MLL-rearranged (MLL-r) AML is approximately 40% [8]. Although dose intensification of chemotherapy might reduce the risk of relapse, it is however associated with the long-term adverse effects and a high rate of treatment-related mortality [9]. Therefore, a more effective and less toxic therapy is urgently needed to treat this subset of AML with poor prognosis.

Chidamide, a novel histone deacetylase (HDAC) inhibitor of the benzamide class that specifically inhibits HDAC1-3, has approved by the Chinese FDA for treatment of relapsed or refractory peripheral T cell lymphoma (PTCL) [10, 11]. Recently, several groups including ours have demonstrated that chidamide displays promising activity against various cancer types, especially hematological malignancies including AML [12–14]. It has been well established that HDACs, of which at least 18 members have been characterized, play the key roles in the epigenetic regulation of gene expression through chromatin remodeling by inhibiting histone deacetylation [15, 16]. Among them, HDAC1, 2 and 3 are frequently overexpressed in human leukemia [17], which could interact with MLL fusion partners and result in aberrant regulation of chromatin remodeling and thus the expression of tumor-driven genes [18]. Earlier studies have demonstrated that chidamide synergizes with conventional chemotherapeutic agents in human leukemia cells by disrupting cell cycle progression and DNA damage responses, as well as promoting ROS-dependent apoptosis [19]. However, because the single-agent activity of chidamide towards relapsed or refractory AML appears not to be satisfied, the combination strategy involving chidamide thus warrants investigation in this disease.

Menin, which is encoded by the multiple endocrine neoplasia 1 (MEN1) gene, is known to interact directly with the N-terminal domain of MLL. This interaction is required for the activation of target gene expression by the MLL-fusion proteins, thereby critical for their capability to mediate transformation [20]. Menin is also involved in DNA damage response (DDR), particularly DNA repair. For example, Menin is accumulated with CHK1 at the sites of double-strand break (DSB) [21]. Thus, the menin-MLL interaction has recently been considered as a potential therapeutic target in MLL-r leukemia [22]. In this context, it raises a possibility that the specific inhibitors targeting the menin-MLL interaction may block MLL fusion protein-mediated leukemic transformation by down-regulating the expression of MLL target genes, an event that is required for the oncogenic activity of MLL fusion proteins. Indeed, this type of small molecule inhibitors has displayed a single-agent anti-proliferative activity in vitro and in vivo in a model of MLL-r leukemia [23]. However, for most targeted agents, the single-agent therapy is unlikely to be curative in AML, primarily due to co-existing perturbations involving multiple leukemogenic signaling pathways in this highly heterogeneous disease. It is therefore anticipated that menin-MLL interaction inhibitors might be more effective in rational combination regimens.

To this end, we sought to examine whether and how the menin-MLL interaction inhibitor MI-3, which acts to inhibit transcription of MLL target genes, would interact with the HDAC inhibitor chidamide, a class of epigenetic agents that regulate gene expression through chromatin remodeling, in MLL-r AML. Here, we reported that simultaneous inhibition of both HDAC and MLL-menin interaction exhibits a synergistic cytotoxic activity in vitro specifically against AML cells carrying MLL-rearrangement. This combination regimen was also active in vivo (e.g., delayed tumor progression and reduced tumor burden) in an MLL-r AML xenograft mouse model. Together, these findings argue that this novel regimen rationally combining the HDAC inhibitor chidamide and the MLL-menin inhibitor MI-3 might represent a promising option for treatment of MLL-r AML.

## Results

### Chidamide synergistically interacts with MI-3 to suppresses the growth of MLL-rearrangement AML cells

**Fig. 1:**
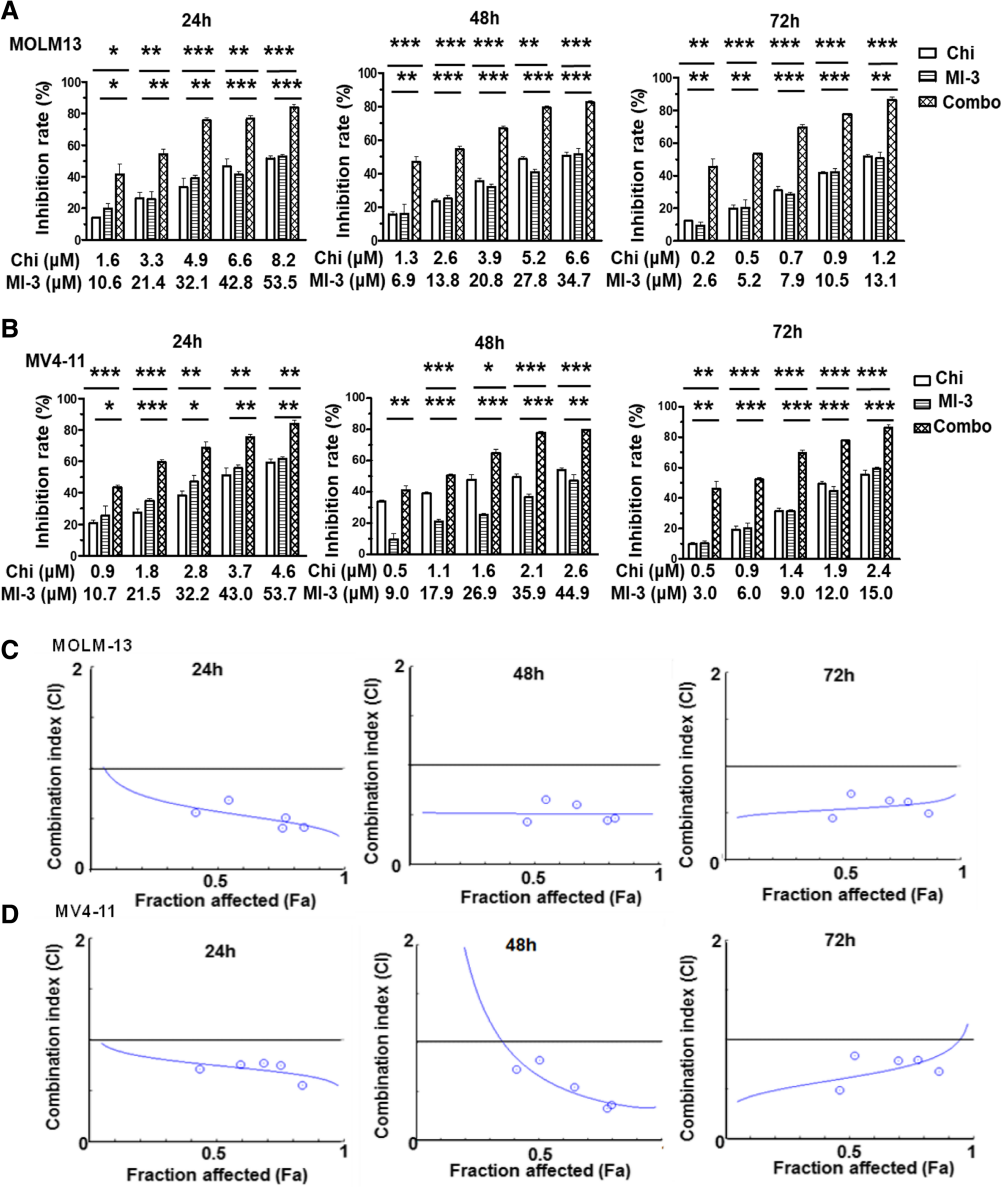
The HDAC inhibitor chidamide synergistically interacts with the menin-MLL interaction inhibitor MI-3 to inhibit cell viability in MLL-r AML cells. **a**, **b** Human MLL-rearranged AML cell lines MOLM-13 (**a**) and MV4-11 cells (**b**) were exposed to the indicated concentrations (μM) of chidamide ± MI-3 (μM) for 24, 48, and 72 h, after which cell viability was examined using the CCK-8 assay. Values indicate mean ± SEM for at least three independent experiments performed in triplicate. *P < 0.05, **P < 0.01, and ***P < 0.001 for comparison with each single agent. **c**, **d** MOLM-13 (**c**) and MV4-11 (**d**) cells were treated as described in Additional file 2: Supplemental Table 1, followed by the analysis of cell viability as above, after which the CompuSyn analysis was performed to determine whether the interaction between these two agents is synergistic (CI value < 1.0)

We first examined the activity of chidamide and MI-3 alone and in combination to determine whether these two agents would synergistically interact with each other in inhibition of MLL-r AML cell viability. To this end, after exposing to a series of concentrations of chidamide or MI-3 for 24, 48, and 72 h, viability of MOLM-13 and MV4-11 cells, both lines carrying MLL-rearrangement, were determined by the CCK-8 analysis. As shown in Additional file 1: Figure S1A–D, exposure to either agent resulted in a marked increase in cytotoxicity towards these MLL-r AML cells in a dose- and time-dependent manner. Based on the single-agent activity after treated for 24, 48, and 72 h, the IC_50_ values for chidamide and MI-3 were calculated in MOLM-13 and MV4-11 cells, respectively (Table 1). Of note, the combined treatment with subtoxic doses of chidamide and MI-3 (≤ IC50 for each agent) resulted in a sharp increase in inhibition rate of cell viability in MOLM-13 (Fig. 1a) and MV4-11 cells (Fig. 1b) at 24, 48, and 72 h. Moreover, the combination index (CI) < 1.0 indicated a synergistic interaction between chidamide (1.6–8.2 μM) and MI-3 (10.6–53.5 μM) in MOLM-13 (Fig. 1c), as well as between chidamide (0.9–4.6 μM) and MI-3 (10.7–53.7 μM) in MV4-11 (Fig. 1d). Values of fraction affected (Fa) and CI for each cell line after treatment for 24, 48, and 72 h were provided in Additional file 2: Table S1, of which the CI values ranged from 0.4 to 0.8. In contrast, the human AML cell lines Kasumi-1 and KG1α without MLL-rearrangement were much less sensitive to this regimen combining chidamide and MI-3 in the same range of concentrations **(**Additional file 1: Figure S2A–C). Together, these results indicate that chidamide synergistically interacts with MI-3 to reduce the viability of AML cells carrying MLL-rearrangement. They also raise a possibility that AML cells carrying MLL-rearrangement might be particularly susceptible to this combination regimen.

**Table 1:** IC50 values of chidamide and MI-3 as single agent in AML cells

		Chidamide		MI-3	
		IC50(μM)	95%CI	IC50(μM)	95%CI
	24 h	8.240	4.868–13.950	53.54	33.920–84.510
MOLM-13	48 h	6.578	4.782–9.050	34.69	23.240–51.790
	72 h	1.180	0.332–4.192	13.11	7.502–22.920
	24 h	4.623	3.822–5.593	53.72	29.770–96.910
MV4-11	48 h	2.630	1.438–4.810	44.90	39.250–51.370
	72 h	2.379	1.274–4.443	15.01	9.520–23.680

### Co-exposure to chidamide and MI-3 induces apoptosis of MLL-rearrangement AML cells, in association with increased ROS generation and mitochondrial injury

**Fig. 2:**
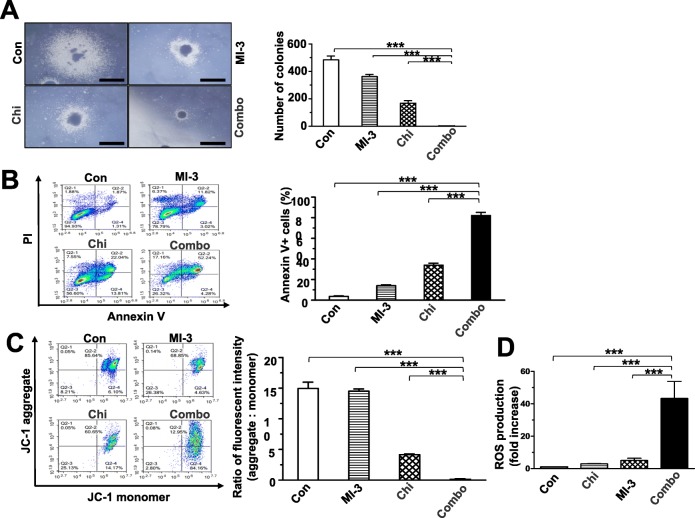
Co-treatment with chidamide and MI-3 induces robust apoptosis, in association with increased ROS generation and loss of mitochondrial membrane potential. **a** MOLM-13 cells were treated with 2.63 μM chidamide ± 13.88 μM MI-3 for 48 h, followed by the colony formation assay. **b**, **c** MOLM-13 cells were treated as above, after which flow cytometric analysis was performed to determine percentage of Annexin V^+^ apoptotic cells (**b**) and cells with loss of mitochondrial membrane potential (**c**). **a**–**c** The representative results were shown (left), and the data for at least three independent experiments was presented as bar graphs (right). **d** In parallel, intracellular ROS levels were measured by flow cytometry using the Reactive Oxygen Species Assay Kit. Values indicate mean ± SEM for at least three independent experiments performed in triplicate (***P < 0.001)

To validate the synergistic effect of the regimen combining chidamide and MI-3 on MLL-r AML cells, the colony formation assay was performed. As shown in Fig. 2a, whereas chidamide (2.6 μM) and MI-3 (13.9 μM) displayed moderate single-agent activity, a significant reduction in colony formation was observed in MOLM-13 cells after combined treatment, compared with these two agents alone. Analogous results were obtained from MV4-11, another MLL-r AML cells (Additional file 1: Figure S3A). Moreover, flow cytometry with Annexin V/PI staining was then performed to examine whether chidamide would interact with MI-3 to induce apoptosis in MLL-r cells. After exposing to chidamide and MI-3 alone or in combination for 48 h, the percentage of apoptotic (Annexin V-positive) cells was significantly increased in MOLM-13 (Fig. 2b) and MV4-11 cells (Additional file 1: Figure S3B), compared to each single agent. As loss of mitochondrial membrane potential (MMP) plays a crucial role in the initiation of intrinsic mitochondrion-dependent apoptotic cascade [25], we next examined the effect of chidamide and MI-3 individually or in combination on MMP. Consistent with the results for apoptosis, combined treatment with chidamide and MI-3 also induced loss of MMP, reflected by impaired mitochondrial depolarization indicated by markedly decreased fluorescence intensity ratio between JC-1 aggregate and monomer (Fig. 2c and Additional file 1: Figure S3C). To unveil the potential mechanism underlying the synergistic interaction between these two agents in the induction of apoptosis, flow cytometry was carried out to monitor intracellular ROS levels. After co-treated with chidamide and MI-3 for 48 h, a significant increase in ROS generation was observed in MOLM-13 (Fig. 2d) and MV4-11 cells (Additional file 1: Figure S3D), compared to treatment with each single agent. Together, these results suggest that chidamide interacts synergistically with MI-3 to induce apoptosis of AML cells carrying MLL-rearrangement via promotion of ROS production and mitochondrial damage.

### Co-treatment with chidamide and MI-3 alters genome-wide gene expression in MLL-rearrangement AML cells

**Fig. 3:**
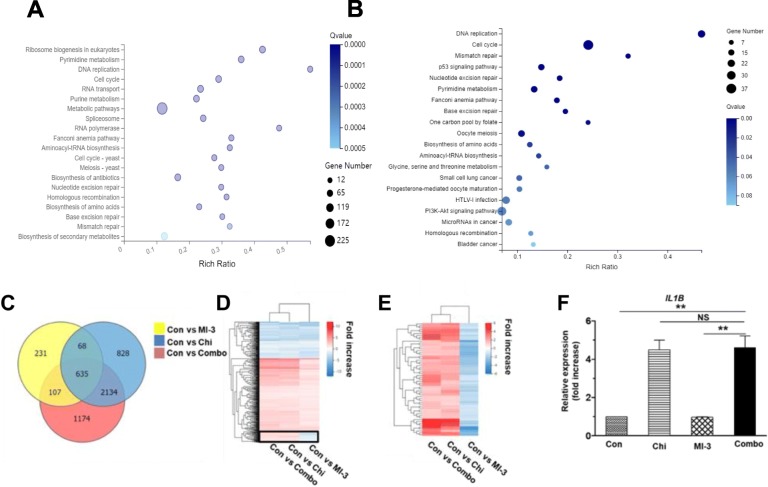
RNA sequencing reveals genome-wide gene expression profiles of MOLM-13 cells treated with chidamide and MI-3 alone or in combination. **a**, **b** MOLM-13 cells were treated with 2.6 μM chidamide ± 13.9 μM MI-3 for 24 h (**a**) or 48 h (**b**), after which total RNA was extracted and subjected to whole exome RNAseq that was performed in triplicate for each condition. The KEEG analysis reveals the annotations of the most enriched pathways of differentially expressing genes after combined treatment for 48 h, compared to untreated control. **c–e** Alternatively, a Venn diagram shows the number of genes and their relationship that were differentially expressed (log2FC ≥ 1, *Q* value ≤ 0.001) after treatment with chidamide and MI-3 alone or in combination, compared to untreated control (**c**). A heatmap shows hierarchical clustering of 635 genes significantly affected by all three treatments, including chidamide, MI-3, and combined treatment (**d**). A heatmap shows hierarchical clustering of 59 genes (indicated by square in panel B) that were upregulated by chidamide and combined treatment, but downregulated by MI-3 (**e**). **f** Real-time PCR analysis was performed to monitor expression of *IL1B*, one of 59 genes shown in panel **c**, in MOLM-13 cells after treated with chidamide ± MI-3 for 48 h. The reaction was carried out in triplicate and relative expression levels were calculated as 2^−△△CT^ after normalization to β-actin. Values indicate mean ± SEM for at least three independent experiments performed in triplicate (**P < 0.01, NS = not significant)

Anti-tumor activity of both HDAC inhibitor and menin-MLL interaction inhibitor involves transcriptional regulation of gene expression [26–29]. To further understand the mechanism of action underlying the synergistic interaction between chidamide and MI-3 in AML-carrying MLL-rearrangement, the RNAseq assay was then performed to profile genome-wide gene expression in MOLM-13 cells after treated with chidamide and MI-3 alone or in combination (see Additional file 2: Table S2 for a list of all genes). After combined treatment with chidamide and MI-3 for 24 h (Fig. 3a) and 48 h (Fig. 3b), the KEGG analysis indicated that the most significantly altered pathways involving cell cycle, DNA replication, and several DNA repair mechanisms [e.g., homologous recombination (HR), nucleotide excision repair, base excision repair, mismatch repair, and Fanconi anemia pathway]. Further, the GESA analysis further revealed that most of these alterations stemmed from chidamide, rather than MI-3 (Additional file 1: Figure S4A–C). Moreover, the analysis of the dataset (48 h treatment) for the significantly downregulated or upregulated transcripts (log2FC ≥ 1, *Q* value ≤ 0.001) revealed gene expression profile (GEPs) for chidamide (blue) and MI-3 (yellow) alone or in combination (red) with an overlap of 635 genes (Fig. 3c). A majority of these overlapped genes displayed the same trends of expression (i.e., up- or downregulation) in MOLM-13 cells treated with MI-3 and chidamide alone or in combination (Fig. 3d). However, there was a small cluster of 59 genes (indicated by square in Fig. 3d) that were differentially expressed in MOLM-13 cells exposed to MI-3 (downregulation) versus chidamide alone or in combination (upregulation; Fig. 3e). The gene ontology (GO; Additional file 1: Figure S4D) and KEGG (Additional file 1: Figure S4E) analyses revealed that these genes were associated with several key survival signaling pathways (e.g., MAPK, NF-κB). They also suggested that cytokine signaling pathways (e.g., TNF), which is essential for the inflammatory reaction [30] and known to be involved in single-agent activity of chidamide [31], might also be involved in the interaction between chidamide and MI-3 in MLL-r AML cells. In this context, the real-time PCR analysis was performed to validate the expression of *IL1B* that encodes the pro-inflammatory cytokine IL-1β, a representative gene selected from 59 overlapping genes described in Fig. 3e. Consistent with the RNAseq results, exposure of MOLM-13 cells to chidamide in the absence or presence of MI-3 resulted in a marked increase in expression of *IL1B* (Fig. 3f). However, qPCR failed to detect whether MI-3 alone downregulated the expression of *IL1B*, due to its low basal level in untreated cells. Together, these results suggest that the mechanism of action underlying anti-leukemic activity of the combination treatment with chidamide and MI-3 might involve DDR in MLL-r AML cells. They also raise the possibility that co-administration of chidamide might reactivate a set of genes that are silenced by MI-3.

### Co-treatment with chidamide and MI-3 disrupts DNA damage response and results in DNA damage in AML-carrying MLL-rearrangement

**Fig. 4:**
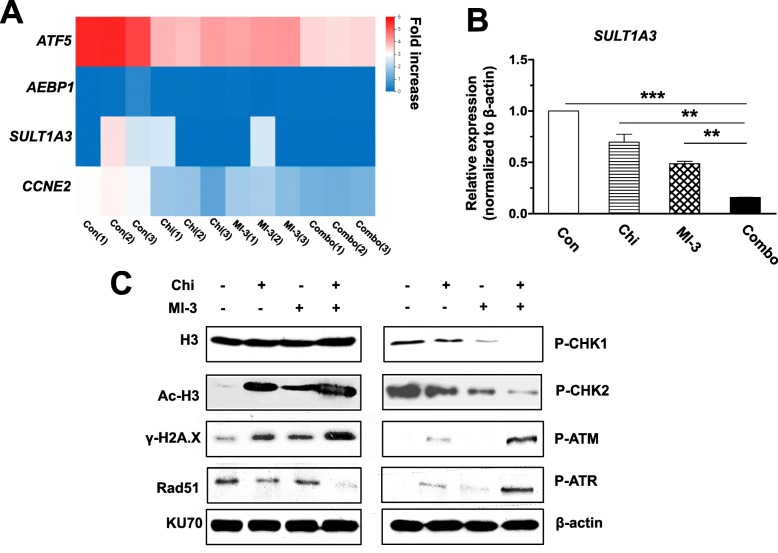
Combined treatment with chidamide and MI-3 disrupts DNA damage response. **a **A heatmap shows hierarchical clustering of four genes, among 635 genes described in Fig. 3a, that were downregulated ≥ 2-folds by chidamide or MI-3 alone and ≥ 4-fold by these two agents in combination. **b** Real-time PCR analysis was performed to validate the expression of *SULT1A3*, one of 4 genes shown in panel **a**, in MOLM-13 cells treated with chidamide ± MI-3 for 48 h. The reaction was carried out in triplicate and relative expression levels were calculated as 2^−△△CT^ after normalization to β-actin. Values indicate mean ± SEM for at least three independent experiments performed in triplicate (**P < 0.01 and ***P < 0.001). **c** MOLM-13 cells were exposed to chidamide ± MI-3 for 48 h, after which western blot analysis was performed to monitor the expression of the indicated proteins involved in DNA damage checkpoint and DNA repair, as well as γH2A.X, a marker for DNA double-strand breaks

We then performed a gene set enrichment analysis (GSEA) of differentially expressing genes in MOLM-13 cells after treated with chidamide and MI-3 alone or in combination. Four genes were identified, which met both of the following criteria, including (a) at least 2-fold downregulation by chidamide or MI-3 and (b) at least 4-fold downregulation by combined treatment. These genes included *SULT1A3* (sulfotransferase 1A3/1A4), *AEBP1* (transcriptional repressor), *CCNE2* (cyclin E2), and *ATF5* (transcription factor; Fig. 4a and Additional file 1: Figure S5). Among them, *SULT1A3* is known to catalyze sulfation of nitrotyrosine, which is associated with DNA damage [32, 33]. Since the RNAseq analysis after treatment for either 24 or 48 h (Additional file 1: Figure S4A and S4B) suggested a potential role of DNA damage response (DDR) in the anti-tumor activity of the regimen combining chidamide and MI-3, qPCR analysis was performed to validate expression of *SULT1A3* as representative. Consistent with the results of the RNAseq analysis (Fig. 4a), exposure to either chidamide or MI-3 was able to downregulate *SULT1A3* expression, while the combination treatment resulted in a greater reduction in its expression (Fig. 4b). In this context, western blot analysis was further carried out to examine the DDR signaling pathway, which is orchestrated by ATM and ATR as well as their key downstream checkpoint kinases CHK1 and CHK2. As shown in Fig. 4c and Additional file 1: Figure S6, exposure to chidamide in the presence or absence of MI-3 led to increased acetylation of histone H3 in various AML cell lines, due to inhibition of its deacetylation catalyzed by HDACs. Interestingly, treatment with MI-3 also modestly increased H3 acetylation in MOLM-13 cells (Fig. 4c), but not in MV4-11 or Kasumi-1 cells (Additional file 1: Figure S6), suggesting a cell line-specific phenomenon. Notably, combined treatment with these two agents clearly induced phosphorylation (activation) of both ATM and ATR. In contrast, whereas exposure to either MI-3 or in a lesser extent chidamide attenuated phosphorylation (activation) of both CHK1 and CHK2, these events were almost completely inhibited by co-administration of these two agents (Fig. 4c). Combined treatment also markedly downregulated the expression of Rad51, an important DNA repair protein [34], while did not affect the levels of another DNA repair protein KU70. As a consequence, the combination treatment induced robust DNA damage, manifested by a sharp increase in expression of γH2A.X (Fig. 4c), a marker of DNA double-strand break [35]. Together, these results suggest that disruption of the DNA damage checkpoint through inactivation of CHK1 and CHK2, rather than their upstream kinases ATM and ATR, as well as interfere with the DNA repair machinery by downregulating DNA repair proteins (e.g., Rad51), might account for or at least contribute to the synergistic interaction between chidamide and MI-3 in AML cells carrying MLL-rearrangement.

### The regimen combining chidamide and MI-3 is active in vivo in a xenograft model of AML-carrying MLL-rearrangement

**Fig. 5:**
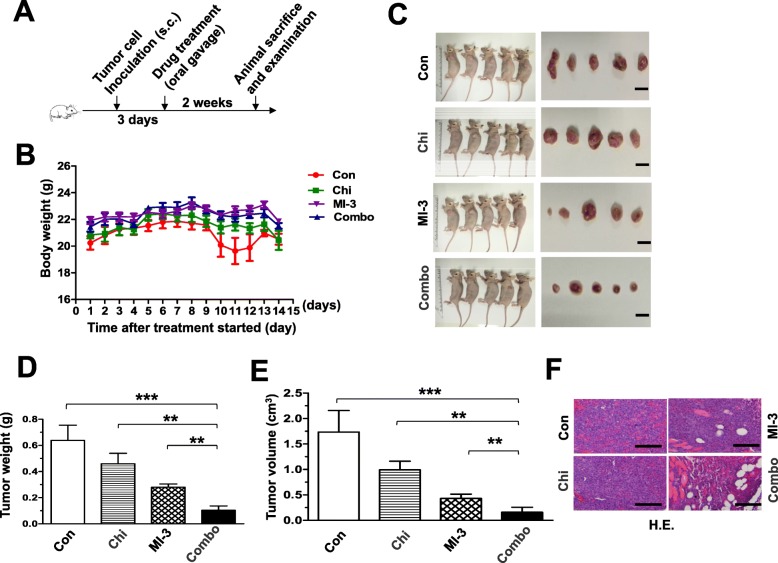
Co-administration of chidamide and MI-3 suppresses tumor growth in a xenograft model of MLL-r AML. **a** A scheme for the animal study that are described in detail in the “Materials and methods” section. **b** Mice were weighed daily after tumor cell inoculation. **c–e** Mice were sacrificed after drug treatment for 14 days, and then the images of mice and tumors were captured (**c**). Weight (**d**) and volume (**e**) of tumors were measured and calculated. Values indicate mean ± SEM for 6 mice/each group (**P < 0.01, ***P < 0.001). **f** Tumors sections were prepared and stained by H&E for histological examination

Last, anti-tumor activity of the regimen combining chidamide and MI-3 was examined in a mouse xenograft model established by subcutaneous inoculation with MLL-r MOLM13 cells. After 3 days, mice were randomly divided into four groups, including vehicle control, chidamide, MI-3, and the combination treatment (Fig. 5a). Although a moderate reduction in body weight was observed at days 10–12 after treatment with chidamide and MI-3 in combination, but rapidly recovered at day 14 (Fig. 5b). Otherwise, no other signs of notable toxicity were observed. Significantly, combined treatment with chidamide and MI-3 resulted in a marked reduction in tumor burden, reflected by decreased volume and weight of tumor masses, compared to vehicle control as well as each individual agent (Fig. 5c–e). These results were also confirmed by histological examination (Fig. 5f). Together, these findings indicate that the combination regimen of chidamide and MI-3 is effective in vivo against MLL-r AML, while well tolerated.

### Discussion

AML is a highly heterogeneous disease, of which 35–50% in infant and ~ 10% in adult carry MLL-rearrangement [2–4], a genetic abnormality that leads to various MLL fusion proteins with strong oncogenic property [36]. This subset of AML is particularly aggressive and often associated with poor prognosis, primarily due to the lack of effective treatment [37, 38]. Menin acts as a key cofactor of oncogenic MLL fusion proteins [39]. For example, menin is required for the maintenance of HOX gene expression mediated by MLL fusion proteins [28]. In this context, small molecule inhibitors targeting the interaction between menin and MLL fusion proteins are recently emerging to treat AML carrying MLL-rearrangement. Indeed, these inhibitors are able to reverse MLL fusion protein-mediated leukemic transformation via downregulation of HOX genes. Therefore, this novel class of anti-cancer agents has recently attracted a lot of interests in treatment of poorly prognostic AML-bearing MLL-rearrangement. However, although earlier studies have demonstrated promising activity of several menin-MLL interaction inhibitors in vivo in MLL-r AML xenograft mouse models, therapeutic responses remain limited. To this end, our observations in the present study indicate that a rationale-based regimen combining these inhibitors (e.g., MI-3) and HDAC inhibitors (e.g., chidamide) might display significantly increased anti-tumor activity towards AML carrying MLL-rearrangement, compared to each single agent, at least in the preclinical setting.

The rationale for combining inhibitors of HDAC and menin-MLL interaction is laid on their properties targeting transcription-regulatory machinery. On the one hand, HDACs regulate gene expression via chromatin remodeling [26]. They function to “tighten” or “close” chromatin structure by deacetylating nucleosomal histones (e.g., H3 and H4) to reduce the accessibility of transcription factors [27]. In contrast, HDAC inhibitors act to “loose” or “open” chromatin structure through inhibition of histone deacetylation by HDACs, which allows transcription factors to access and binding to promoters of target genes, thereby initiating and promoting their expression [41–43]. Notably, the implication of HDACs in AML has promoted the attempt to use the HDAC inhibitors to treat this disease [44, 45]. Moreover, leukemia-carrying MLL-rearrangement is highly susceptible to HDAC inhibition [46]. On the other hand, MLL methylates histone H3 to upregulate expression of target genes, including several HOX genes [28, 29]. In MLL-rearranged AML, certain MLL fusion proteins display enhanced activity to promote gene transcription by recruiting a transcriptional activation complex known as P-TEFb that consists of CDK9 and cyclin T1 [47, 48]. The leukemia-driving activity of MLL fusion proteins relies on their interaction with menin, a protein encoded by the multiple endocrine neoplasia (MEN1) gene [49]. Menin directly binds to the N-terminal domain of MLL in virtually all MLL fusion proteins, an event that is essential for leukemic transformation [50]. In this context, small molecule inhibitors (e.g., MI-3) specifically targeting the interaction between menin and MLL fusion proteins have recently been emerging to treat acute leukemias harboring MLL-rearrangement [51]. Therefore, as the mechanisms of action for both HDAC and menin-MLL interaction inhibitors involve transcription-regulatory machinery, a possibility then arose that these two classes of agents may interact synergistically in MLL-rearranged leukemia. Indeed, the results of the present study demonstrated a highly synergistic interaction between chidamide and MI-3 at their subtoxic dose ranges in human AML cells carrying MLL-rearrangement (e.g., MOLM-13 and MV4-11), but not in cells that did not harbor this genetic abnormality. The anti-tumor activity of this combination regimen was further validated in vivo in a mouse xenograft model of human MLL-r AML. In addition, marked inhibition of the colony-forming activity of MLL-r AML cells by the combination treatment raises the possibility that this regimen might also suppress self-renewal of leukemic stem cells.

Because both HDAC and menin-MLL interaction inhibitors target general transcription-regulatory machinery [40, 52], they may thus affect genome-wide expression of numerous target genes. To this end, the RNAseq analysis revealed that exposure to chidamide and MI-3 alone influenced expression of 3665 and 1041 genes in MLL-r AML cells, respectively, while combined treatment resulted in altered expression of 4050 genes. Moreover, although treatment with chidamide and MI-3 alone or in combination led to global changes in gene expression, a set of 635 genes that were overlapped among these three treatment conditions was observed. Among them, a small cluster of 59 genes was oppositely regulated by MI-3 and chidamide (either alone or in combination). The functions of these differentially expressed genes were involved in several signaling pathways (e.g., MAPK, NF-κB) crucial for tumor cell survival and proliferation [30, 53]. In addition, the roles of these genes also involved the inflammatory responses (e.g., the TNF-signaling pathway). However, the effect of the combination treatment on inflammatory responses (e.g., expression of IL-1β, a well-established marker of inflammation) [54, 55] seems to solely stem from chidamide, rather than MI-3, thereby arguing against that this effect represents the primary mechanism for the high synergy between these two agents. Therefore, an alternative strategy for further analysis of this RNAseq dataset might be required to identify other candidate pathways and targets responsible for or involved in the synergistic interaction between chidamide and MI-3 in MLL-r AML.

In the present study, bulk evidence indicates that the anti-tumor activity of the regimen combining chidamide and MI-3 towards MLL-r AML was associated with induction of apoptosis, primarily via the intrinsic mitochondrion-dependent pathway [36, 56]. First, while exposure to chidamide led to the loss of mitochondrial membrane potential, reflecting mitochondrial injury [57, 58], this event was significantly potentiated by co-administration of MI-3. Second, as mitochondria are considered as the main source of ROS in the cell [59, 60], a robust increase in ROS generation was observed in MLL-r AML cells exposed to both chidamide in the presence of MI-3. Last, flow cytometric analysis revealed that whereas treatment with either chidamide or MI-3 moderately increased apoptotic (Annexin V-positive) cells, this event was dramatically potentiated after co-administration of these two agents. Therefore, these observations argue strongly that chidamide might interact with MI-3 to activate the mitochondrion-related apoptotic signaling cascade. However, due to the multifacial functions of HDAC inhibitors, a caveat that other mechanisms might also contribute to the anti-leukemia effect of chidamide alone and even in combination with MI-3 in MLL-r AML cells could not be excluded.

Both menin and HDAC also play important roles in DNA damage response (DDR), particularly including DNA damage checkpoint and DNA repair [61, 62]. Moreover, it has been reported that HDAC or menin-MLL interaction inhibitors could induce apoptosis through interference with cytoprotective DDR [61, 63]. Thus, it raises a possibility that these two classes of agents may interact to disrupt DDR and therefore trigger DNA damage in MLL-r AML cells. Indeed, co-exposure to chidamide and MI-3 led to a sharp increase in S139 phosphorylation of H2A.X (termed γH2A.X), a well-established marker for DNA double-strand breaks (DSB). Interestingly, co-administration of chidamide and MI-3-induced phosphorylation (activation) of ATM and ATR, two kinases involved in initiation of DDR [64, 65]. However, combined treatment almost completely inhibited phosphorylation (activation) of CHK1 and CHK2, two key kinases of DNA damage checkpoint that act as direct downstream targets of ATR and ATM [66, 67]. Taken together, these results suggest that this combination regimen appears to target primarily on CHK1 and CHK2, rather than their upstream kinases (ATR and ATM), while activation of the latter might reflect a feedback response to inactivation of CHK1 and CHK2 after co-exposing to chidamide and MI-3. In addition, the combined treatment also downregulated Rad51, a DNA repair protein that plays a major role in homologous recombination (HR) repair of DSB [34]. However, it did not affect the levels of Ku70, another DNA repair protein critical for non-homologous end joining (NHEJ) repair of DSB [68]. These observations suggest that the regimen combining chidamide and MI-3 might selectively target DNA repair via HD, rather than NHEJ. Interestingly, the GSEA analysis of the RNAseq dataset identified *SULT1A3* as one of four genes that were downregulated by chidamide and MI-3 alone or in combination, which was confirmed by real-time PCR analysis. *SULT1A3* encodes an enzyme involved in the metabolism of nitrotyrosine, while increased levels of nitrotyrosine serve as a biomarker of oxidative stress that induces DNA damage [32, 33]. Thus, this finding raises the possibility that *SULT1A3* might represent a potential target that links oxidative stress (e.g., ROS) and DNA damage together in MLL-r AML cells co-treated with MI-3 and HDAC inhibitors. However, further studies are required to address this possibility.

In summary, the findings of the present study demonstrate a highly synergistic interaction between the HDAC inhibitor chidamide and the menin-MLL interaction inhibitor MI-3 both in vitro and in vivo in AML cells with MLL-rearrangement. They also provide evidence for the potential mechanisms underlying the markedly increased anti-leukemia activity of this combination regimen, including ROS generation, apoptosis induction via the mitochondrion-dependent signaling pathway, and disruption of DDR (e.g., DNA damage checkpoint and DNA repair via HR). In addition, the genome-wide gene expression profile (GEP) by the RNAseq analysis might serve as a resource for future studies to identify potential targets (e.g., *SULT1A3*) and pathways to further understand the mechanisms of action for these two classes of agents in AML carrying MLL-rearrangement. Due to the current lack of effective therapy for the treatment of MLL-rearranged AML, the strategy combining HDAC and menin-MLL interaction inhibitors warrants further investigation in this poorly prognostic subset of AML.

## Material and methods

### Reagents

The HDAC inhibitor chidamide (CS055, purity > 95%) was kindly provided by Chipscreen Bioscience Ltd. (Shenzhen, China). The MLL-menin interaction inhibitor MI-3 (purity = 99.03%; Cat. No. S7619) was purchased from Selleck Chemicals (Houston, TX, USA). The reagents were dissolved in dimethyl sulfoxide (DMSO; Invitrogen, Carlsbad, CA, USA) as 10 mM stock solution and stored at − 20 ^o^C, which was then diluted to the required concentrations with cell culture medium prior to experiments.

### Cell culture and sorting

Human AML cell lines MOLM-13 and MV4-11 carrying MLL-rearrangement were purchased from ATCC (Rockefeller, MD, USA) and cultured at 37 °C in a 5% CO_2_ incubator in Iscove’s modified Dulbecco’s medium (IMDM) and RPMI-1640 medium (HyClone, Thermo Scientific, Logan, UT, USA) supplemented with 10% fetal bovine serum (FBS, Gibco, Thermo Scientific, Grand Island, NY, USA), respectively. For sorting CD34^+^/CD38^−^ cells, cells were stained with hCD34-APC (eBioscience, Thermo Scientific, San Diego, CA, USA) and hCD38-PE (eBioscience, Thermo Scientific, San Diego, CA, USA) for 30 min at 4 °C. After washed twice with PBS containing 1% FBS, CD34^+^/CD38^−^ cells were sorted using FACS Aria IIU (Biosciences, Franklin Lakes, NJ, USA).

### Analysis of cell proliferation and viability

Cytotoxicity was determined by using the Cell Counting Kit-8 (CCK-8, Dojindo, Kumamoto, Japan) [24]. Briefly, 2 × 10^4^/well cells were seeded in 100 μl medium on 96-well plates and treated with indicated concentrations of chidamide and MI-3 alone or in combination for 24, 48, and 72 h. The CCK-8 reagent (10 μl/well) was then added and incubated for additional 2 h, after which the absorbance at 450 nm was detected by a microplate reader (ELx800, BioTek, Winooski, VT, USA). The data from three independent experiments in triplicate was presented as percentage of viable cells by comparing to untreated control. IC50 values were determined using the SPSS 20.0 software.

### Analysis of apoptosis

Cells were cultured and treated chidamide with or without MI-3 for 24, 48, or 72 h as described above, followed by double staining with Annexin V-FITC and PI (eBioscience, Thermo Scientific, San Diego, CA, USA) for 15 min at room temperature in dark as per the manufacturer’s instruction. Cells were then analyzed by flow cytometry (FACS Fortessa, BD Biosciences, Franklin Lakes, NJ, USA). The percentage of Annexin V-positive (apoptotic) cells was determined.

### Analysis of mitochondrial membrane potential

2 × 10^5^/ml cells were seeded in 24-well plates and treated with chidamide and MI-3 alone or in combination at indicated concentrations. After 24 h incubation, cells were harvested and stained with 2 μM Rhodamine 123 (Byeotime, Shanghai, China) for 30 min at 37 ^o^C. After washed, mitochondrial membrane potential (MMP) was analyzed by flow cytometry (FACS Fortessa).

### Measurement of ROS

Intracellular ROS levels were determined using the Reactive Oxygen Species Assay Kit (no. S0033, Beyotime, Shanghai, China) as per the manufacturer’s instruction. Briefly, after treated with indicated concentrations of chidamide and MI-3 alone or in combination for 24 h, cells were harvested and stained with 10 μM 2′,7′-dichlorofluorescein diacetate (DCFH-DA, 1:1000 diluted in RPMI1640) for 20 min at 37 °C. After washed twice, intracellular ROS level was determined by flow cytometry (FACS Fortessa).

### Colony formation assay

2×10^5^/well cells at logarithmical growth phase were seeded in 24-well plates and treated with indicated concentrations of chidamide and MI-3 alone or in combination for 48 h. After washed, 500/well cells were then cultured in complete methylcellulose medium (R&D Systems, Minneapolis, MN, USA) in 3.5 cm dishes for 14 days. Colonies consisting of at least 50 cells were counted and analyzed for colony-forming capability.

### Western blot analysis

2×10^5^/well cells were treated with chidamide in the absence or presence of and/or MI-3 for 48 h, and the subjected to western blot analysis using indicated primary antibodies and secondary HRP-conjugated antibodies (1:10,000, Abcam, Cambridge, UK). The primary antibodies included anti-caspase-3 (#9662S), anti-PARP (#9532S), anti-histone H3 (#4499S), anti-phospho-H3 (#53348S), anti-γH2A.X (#2577S), anti-RAD51 (#8875S), anti-KU70 (#4588S), anti-STAT3 (#9139 S), anti-Mcl-1 (#94296S), anti-phospho-p53 (#9286S), anti-p21(#2947S), anti-phospho-ATM (#5803S), anti-ATM (#2873S), anti-phospho-ATR (#2835S), anti-CHK1 (#2360), anti-CHK2 (#2662), anti-P-CHK1 (#2197S), and anti-P-CHK2 (#2348S) from Cell Signaling Technology (Boston, MA, USA). The primary antibodies were diluted with 5% fat-free milk-TBST. Anti-β-actin (1:1000, Cell Signaling Technology) was used as loading control. Blots were then detected using the ECL Western Blotting Detection Kit (GeneFlow, Staffordshire, UK).

### RNA sequencing

Cells were incubated with chidamide with or without MI-3 for 24 or 48 h, after which total RNA was isolated as described previously [12]. RNA sequencing (RNAseq) was then carried out via a commercially available service (service ID# F18FTSSCWLJ1284, BGI, Huada Gene, Wuhan, China). Briefly, after total RNA was fragmented into short fragments and mRNA was enriched using oligo (dT) magnetic beads, followed by cDNA synthesis. Double-stranded cDNA was purified and enriched by PCR amplification, after which the library products were sequenced using BGIseq-500. The KEGG pathway and GO bioinformatics analyses were performed by the BGI, using the Dr. TOM approach, an in-house customized data mining system of the BGI. Altered (upregulated or downregulated) expression of genes was expressed as log2FC, which represents log-transformed fold change (log2FC = log2[B] − log2[A], while A and B represent values of gene expression for different treatment conditions).

### Quantitative real-time PCR

After total RNA was extracted and mRNA purified, mRNA was converted to cDNA using the TransScriptor First-Strand cDNA Synthesis SuperMix (TransScript, #AT301, Beijing, China). The assays-on-demand primers and probes and TaqMan Universal Master Mix were used to examine gene expression by the Roche LC480 Sequence Detection System (TransStart) according to the user’s manual. The house-keeping gene ACTB (encoding β-actin) was used as internal control. All reactions were carried out in triplicate and relative expression levels were calculated as 2^−△△CT^ after normalization to the internal control. Each sample was analyzed independently three times.

### Animal study

5 × 10^6^ MOLM-13 cells were inoculated subcutaneously into nude mice. After 3 days, mice were randomly divided into four groups (6 mice per group), including vehicle (PBS) control, 30 mg/kg/days chidamide, 40 mg/kg/days MI-3, and both chidamide and MI-3 by intraperitoneal injection and oral gavage, respectively. Tumor size and body weight were measured daily. Mice were sacrificed after drug treatment for 14 days when tumor size in any mice reached 20 mm^3^. All tumors was removed, measured, and weighted. Tumor volume was calculated using the formula *V* = 1/2(*L* × *W*^2^) (L:length, W:width). Histological examination was performed on tumor sections after H&E staining.

### Statistical analysis

Values represent the mean ± SEM for at least three independent experiments. All statistical analyses were carried out using the SPSS 20.0 and GraphPad Prism 5.0 softwares. Variables between two groups were compared using the two-tailed Student’s *t* test. Comparisons among multiple groups were performed using the one-way analysis of variance (ANOVA) followed by the Bonferroni post hoc test. *P* < 0.05 was considered as statistically significant.

## Additional files

Additional file 1: **Figure S1.** The HDAC inhibitor chidamide and the Menin-MLL interaction inhibitor MI-3 display dose- and time-dependent effects on viability of MLL-r AML cells. **Figure S2.** The regimen combining chidamide and MI-3 is not effective in non-MLL-r AML cells. **Figure S3.** Co-treatment with chidamide and MI-3 induces robust apoptosis, ROS generation, and loss of mitochondrial membrane potential in MLL-r MV4-11 cells. **Figure S4.** The KEGG analysis of the RNAseq data reveals involvement of multiple cell cycle and DNA repair pathways in the interaction between chidamide and MI-3 in MLL-r AML cells. **Figure S5.** Genome-wide RNA sequencing identifies four genes that are differentially expressed in MOLM-13 cells treated with MI-3 vs. chidamide alone or in combination. **Figure S6.** Treatment with chidamide results in increased acetylation of histone H3 in both MLL-r and non-MLL-r AML cells. (DOCX 3896 kb)

Additional file 2: **Table S1.** List of all genes that were differentially expressed in cells exposed to MI-3, chidamide, or both, in which 635 overlapped genes shown in the Venn Diagram (Figure 3C) as well as 59 genes indicated by a square (Figure 3D) are highlighted in pink and yellow, respectively. (XLSX 1820 kb)

## Data Availability

The RNAseq datasets of the present study are available on request from the corresponding author.

## Abbreviations

AML: Acute myeloid leukemia
CI: Combination index
DDR: DNA damage response
HDAC: Histone deacetylase
MLL-r: MLL-rearrangement

## References

[1] Kuykendall A, Duployez N, Boissel N, Lancet JE, Welch JS (2018). Acute myeloid leukemia: the good, the bad, and the ugly. American Society of Clinical Oncology educational book. Am Soc Clin Oncol.

[2] Kim HJ, Cho HI, Kim EC, Ko EK, See CJ, Park SY (2002). A study on 289 consecutive Korean patients with acute leukaemias revealed fluorescence in situ hybridization detects the MLL translocation without cytogenetic evidence both initially and during follow-up. Br J Haematol.

[3] Grimwade D, Hills RK, Moorman AV, Walker H, Chatters S, Goldstone AH (2010). Refinement of cytogenetic classification in acute myeloid leukemia: determination of prognostic significance of rare recurring chromosomal abnormalities among 5876 younger adult patients treated in the United Kingdom Medical Research Council trials. Blood..

[4] BV B, Raimondi SC, Harbott J, Zimmermann M, Alonzo TA, Auvrignon A (2009). Novel prognostic subgroups in childhood 11q23/MLL-rearranged acute myeloid leukemia: results of an international retrospective study. Blood..

[5] Marneth AE, Prange KHM, Al Hinai ASA, Bergevoet SM, Tesi N, Janssen-Megens EM (2018). C-terminal BRE overexpression in 11q23-rearranged and t(8;16) acute myeloid leukemia is caused by intragenic transcription initiation. Leukemia..

[6] Muntean AG, Hess JL (2012). The pathogenesis of mixed-lineage leukemia. Ann Rev Pathol..

[7] Meyer C, Hofmann J, Burmeister T, Gröger D, Park TS, Emerenciano M (2013). The MLL recombinome of acute leukemias in 2013. Leukemia..

[8] Zeisig BB, Kulasekararaj AG, Mufti GJ, So CW (2012). SnapShot: acute myeloid leukemia. Cancer cell..

[9] Sheridan C (2017). First new drug approval for AML in 15 years. Nature biotechnology..

[10] Zhang H, Dong L, Chen Q, Kong L, Meng B, Wang H, Fu K (2017). Synergistic antitumor effect of histone deacetylase inhibitor and doxorubicin in peripheral T cell lymphoma. Leukemia Res..

[11] Ellmeier W, Seiser C (2018). Histone deacetylase function in CD4(+) T cells. Nature reviews. Immunology..

[12] Li Y, Wang Y, Zhou Y, Li J, Chen K, Zhang L (2017). Cooperative effect of chidamide and chemotherapeutic drugs induce apoptosis by DNA damage accumulation and repair defects in acute myeloid leukemia stem and progenitor cells. Clinical epigenetics..

[13] Gao S, Li X, Zang J, Xu W, Zhang Y (2017). Preclinical and clinical studies of chidamide (CS055/HBI-8000), An orally available subtype-selective HDAC inhibitor for cancer therapy. Anti Cancer Agents Med Chem..

[14] Shi P, Zhang L, Chen K, Jiang Z, Deng M, Zha J (2017). Low-dose decitabine enhances chidamide-induced apoptosis in adult acute lymphoblast leukemia, especially for p16-deleted patients through DNA damage. Pharmacogenomics..

[15] Blagitko-Dorfs N, Schlosser P, Greve G, Pfeifer D, Meier R, Baude A (2019). Combination treatment of acute myeloid leukemia cells with DNMT and HDAC inhibitors: predominant synergistic gene downregulation associated with gene body demethylation. Leukemia..

[16] Ratner M (2014). Small biotech steers HDAC inhibitor to clinic. Nature biotechnology..

[17] Yang H, Maddipoti S, Quesada A, Bohannan Z, Cabrero Calvo M, Colla S (2015). Analysis of class I and II histone deacetylase gene expression in human leukemia. Leuk Lymphoma..

[18] Chen J, Santillan DA, Koonce M, Wei W, Luo R, Thirman MJ (2008). Loss of MLL PHD finger 3 is necessary for MLL-ENL-induced hematopoietic stem cell immortalization. Cancer research..

[19] Li X, Yan X, Guo W, Huang X, Huang J, Yu M (2017). Chidamide in FLT3-ITD positive acute myeloid leukemia and the synergistic effect in combination with cytarabine. Biomed Pharmacother..

[20] Karnik SK, Hughes CM, Gu X, Rozenblatt-Rosen O, McLean GW, Xiong Y (2005). Menin regulates pancreatic islet growth by promoting histone methylation and expression of genes encoding p27Kip1 and p18INK4c. Proc Natl Acad Sci U S A..

[21] Gallo A, Agnese S, Esposito I, Galgani M, Avvedimento VE (2010). Menin stimulates homology-directed DNA repair. FEBS Letters..

[22] Borkin D, Pollock J, Kempinska K, Purohit T, Li X, Wen B (2016). Property focused structure-based optimization of small molecule inhibitors of the protein-protein interaction between menin and mixed lineage leukemia (MLL). J Med Chem..

[23] Borkin D, Klossowski S, Pollock J, Miao H, Linhares BM, Kempinska K (2018). Complexity of blocking bivalent protein-protein interactions: development of a highly potent inhibitor of the menin-mixed-lineage leukemia interaction. J Med Chem..

[24] Wang R, Zhang S, Chen X, Li N, Li J, Jia R (2018). CircNT5E acts as a sponge of miR-422a to promote glioblastoma tumorigenesis. Cancer Res..

[25] Jarman PJ, Noakes F, Fairbanks S, Smitten K, Griffiths IK, Saeed HK (2019). Exploring the cytotoxicity, uptake, cellular response, and proteomics of mono- and dinuclear DNA light-switch complexes. J Am Chem Soc..

[26] Garrido Castro P, van Roon EHJ, Pinhanços SS, Trentin L, Schneider P, Kerstjens M (2018). The HDAC inhibitor panobinostat (LBH589) exerts in vivo anti-leukaemic activity against MLL-rearranged acute lymphoblastic leukaemia and involves the RNF20/RNF40/WAC-H2B ubiquitination axis. Leukemia.

[27] Shen Y, Wei W, Zhou DX (2015). Histone acetylation enzymes coordinate metabolism and gene expression. Trends Plant Sci..

[28] El Ashkar S, Schwaller J, Pieters T, Goossens S, Demeulemeester J, Christ F (2018). LEDGF/p75 is dispensable for hematopoiesis but essential for MLL-rearranged leukemogenesis. Blood..

[29] Yokoyama A (2015). Molecular mechanisms of MLL-associated leukemia. Int J Hematol..

[30] Xia Y, Shen S, Verma IM (2014). NF-kappaB, an active player in human cancers. Cancer Immunol Res..

[31] Liang K, Volk AG, Haug JS, Marshall SA, Woodfin AR, Bartom ET (2017). Therapeutic targeting of MLL degradation pathways in MLL-rearranged leukemia. Cell..

[32] Yasuda S, Yasuda T, Liu MY, Shetty S, Idell S, Boggaram V (2011). Sulfation of chlorotyrosine and nitrotyrosine by human lung endothelial and epithelial cells: Role of the human SULT1A3. Toxicol Appl Pharmacol..

[33] Yasuda S, Idell S, Liu MC (2007). Generation and release of nitrotyrosine O-sulfate by HepG2 human hepatoma cells upon SIN-1 stimulation: identification of SULT1A3 as the enzyme responsible. Biochem J..

[34] King HO, Brend T, Payne HL, Wright A, Ward TA, Patel K (2017). RAD51 Is a Selective DNA repair target to radiosensitize glioma stem cells. Stem Cell Rep..

[35] Tsukuda T, Fleming AB, Nickoloff JA, Osley MA (2005). Chromatin remodelling at a DNA double-strand break site in Saccharomyces cerevisiae. Nature..

[36] Kühn MW, Armstrong SA (2015). Designed to kill: novel menin-MLL inhibitors target MLL-rearranged leukemia. Cancer Cell..

[37] Grembecka J, He S, Shi A, Purohit T, Muntean AG, Sorenson RJ (2012). Menin-MLL inhibitors reverse oncogenic activity of MLL fusion proteins in leukemia. Nat Chem Biol..

[38] Borkin D, He S, Miao H, Kempinska K, Pollock J, Chase J (2015). Pharmacologic inhibition of the menin-MLL interaction blocks progression of MLL leukemia in vivo. Cancer Cell..

[39] Chen Y, Jones KL, Anastassiadis K, Kranz A, Stewart AF, Grembecka J (2019). Distinct pathways affected by menin versus MLL1/MLL2 in MLL-rearranged acute myeloid leukemia. Exp Hematol..

[40] Xu S, Aguilar A, Xu T, Zheng K, Huang L, Stuckey J (2018). Design of the first-in-class, highly potent irreversible inhibitor targeting the menin-MLL protein-protein interaction. Angewandte Chemie.

[41] Eckschlager T1, Plch J2, Stiborova M3, Hrabeta J (2017). Histone deacetylase inhibitors as anticancer drugs. Int J Mol Sci..

[42] De Souza C, Chatterji BP (2015). HDAC inhibitors as novel anti-cancer therapeutics. Recent Pat Anti-Cancer Drug Discov..

[43] Hull EE, Montgomery MR, Leyva KJ (2016). HDAC Inhibitors as epigenetic regulators of the immune system: impacts on cancer therapy and inflammatory diseases. BioMed Res Int..

[44] Thomas X (2017). Randomized phase II study of clofarabine-based consolidation for younger adults with acute myeloid leukemia in first remission. J Clin Oncol..

[45] Zhou L., Chen S., Zhang Y., Kmieciak M., Leng Y., Li L., Lin H., Rizzo K. A., Dumur C. I., Ferreira-Gonzalez A., Rahmani M., Povirk L., Chalasani S., Berger A. J., Dai Y., Grant S. (2016). The NAE inhibitor pevonedistat interacts with the HDAC inhibitor belinostat to target AML cells by disrupting the DDR. Blood.

[46] Burbury KL (2016). MLL-aberrant leukemia: complete cytogenetic remission following treatment with a histone deacetylase inhibitor (HDACi). Ann Hematol..

[47] Smith E, Lin C, Shilatifard A (2011). The super elongation complex (SEC) and MLL in development and disease. Genes Dev..

[48] Minzel W, Venkatachalam A, Fink A, Hung E, Brachya G, Burstain I (2018). Small molecules co-targeting CKIalpha and the transcriptional kinases CDK7/9 control AML in preclinical models. Cell..

[49] Kim JH, Baddoo MC, Park EY, Stone JK, Park H, Butler TW (2016). SON and its alternatively spliced isoforms control MLL complex-mediated H3K4me3 and transcription of leukemia-associated genes. Mol Cell..

[50] He S, Malik B, Borkin D, Miao H, Shukla S, Kempinska K (2016). Menin-MLL inhibitors block oncogenic transformation by MLL-fusion proteins in a fusion partner-independent manner. Leukemia..

[51] Dafflon C, Craig VJ, Méreau H, Gräsel J, Schacher Engstler B, Hoffman G (2017). Complementary activities of DOT1L and Menin inhibitors in MLL-rearranged leukemia. Leukemia..

[52] Guha M (2015). HDAC inhibitors still need a home run, despite recent approval. Nat Rev Drug Discov..

[53] Sun Y, Liu WZ, Liu T, Feng X, Yang N, Zhou HF (2015). Signaling pathway of MAPK/ERK in cell proliferation, differentiation, migration, senescence and apoptosis. J Recept Signal Transduct Res..

[54] Palomo J, Dietrich D, Martin P, Palmer G, Gabay C (2015). The interleukin (IL)-1 cytokine family—balance between agonists and antagonists in inflammatory diseases. Cytokine..

[55] Striz I (2017). Cytokines of the IL-1 family: recognized targets in chronic inflammation underrated in organ transplantations. Clin Sci (London, England : 1979)..

[56] Xu Y, Zhang P, Liu Y (2017). Chidamide tablets: HDAC inhibition to treat lymphoma. Drugs Today..

[57] Qu X, Yu H, Jia B, Yu X, Cui Q, Liu Z (2016). Association of downregulated HDAC 2 with the impaired mitochondrial function and cytokine secretion in the monocytes/macrophages from gestational diabetes mellitus patients. Cell Biol Int..

[58] Fu M, Shi W, Li Z, Liu H (2016). Activation of mPTP-dependent mitochondrial apoptosis pathway by a novel pan HDAC inhibitor resminostat in hepatocellular carcinoma cells. Biochem Biophys Res Commun..

[59] Shadel GS, Horvath TL (2015). Mitochondrial ROS signaling in organismal homeostasis. Cell..

[60] Sabharwal SS, Schumacker PT (2014). Mitochondrial ROS in cancer: initiators, amplifiers or an Achilles' heel?. Nat Rev Cancer..

[61] Kottemann MC, Bale AE (2009). Characterization of DNA damage-dependent cell cycle checkpoints in a menin-deficient model. DNA Repair..

[62] Long J, Fang WY, Chang L, Gao WH, Shen Y, Jia MY (2017). Targeting HDAC3, a new partner protein of AKT in the reversal of chemoresistance in acute myeloid leukemia via DNA damage response. Leukemia..

[63] Nikolova T, Kiweler N, Krämer OH (2017). Interstrand Crosslink Repair as a Target for HDAC Inhibition. Trends Pharmacol Sci..

[64] Carrassa L, Damia G (2017). DNA damage response inhibitors: Mechanisms and potential applications in cancer therapy. Cancer Treat Rev..

[65] Berger ND, Stanley FKT, Moore S, Goodarzi AA (2017). ATM-dependent pathways of chromatin remodelling and oxidative DNA damage responses. Philos Trans R Soc Lond B Biol Sci..

[66] Qiu Z, Oleinick NL, Zhang J (2018). ATR/CHK1 inhibitors and cancer therapy. Radiother Oncol..

[67] Bolcun-Filas E, Rinaldi VD, White ME, Schimenti JC (2014). Reversal of female infertility by Chk2 ablation reveals the oocyte DNA damage checkpoint pathway. Science..

[68] Zan H, Tat C, Qiu Z, Taylor JR, Guerrero JA, Shen T (2017). Rad52 competes with Ku70/Ku86 for binding to S-region DSB ends to modulate antibody class-switch DNA recombination. Nat Commun..

