# Supplementary material for: Co-inhibition of HDAC and MLL-menin interaction targets MLL-rearranged acute myeloid leukemia cells via disruption of DNA damage checkpoint and DNA repair
Source: Clin Epigenetics. 2019 Oct 7;11:137. doi: 10.1186/s13148-019-0723-0 (PMC6781368; doi:10.1186/s13148-019-0723-0)
Supplement: Supplementary file 1 — Figure S1. The HDAC inhibitor chidamide and the Menin-MLL interaction inhibitor MI-3 display dose- and time-dependent effects on viability of MLL-r AML cells. Figure S2. The regimen combining chidamide and MI-3 is not effective in non-MLL-r AML cells. Figure S3. Co-treatment with chidamide and MI-3 induces robust apoptosis, ROS generation, and loss of mitochondrial membrane potential in MLL-r MV4-11 cells. Figure S4. The KEGG analysis of the RNAseq data reveals involvement of multiple cell cycle and DNA repair pathways in the interaction between chidamide and MI-3 in MLL-r AML cells. Figure S5. Genome-wide RNA sequencing identifies four genes that are differentially expressed in MOLM-13 cells treated with MI-3 vs. chidamide alone or in combination. Figure S6. Treatment with chidamide results in increased acetylation of histone H3 in both MLL-r and non-MLL-r AML cells. (DOCX 3896 kb) [file 13148_2019_723_MOESM1_ESM.docx]

# Co-inhibition of HDAC and MLL-Menin interaction targets MLL-rearranged acute myeloid leukemia cells via disruption of DNA damage checkpoint and DNA repair

Jing Ye, Jie Zha, Yuanfei Shi, Yin Li, Delin Yuan, Qinwei Chen, Fusheng Lin, Zhihong Fang, Yong Yu, Yun Dai, Bing Xu

**Supplemental data**

**Supplemental Figure S1.** **The HDAC inhibitor chidamide and the Menin-MLL interaction inhibitor MI-3 display dose- and time-dependent effects on viability of MLL-r AML cells.** (**A**-**D**) human MLL-rearranged AML cell lines MOLM-13 **(A, B)** and MV4-11 cells **(C, D)** were exposed to the indicated concentrations (μM) of chidamide (**A, C**) or MI-3 (**B, D**) for 24 - 72 hrs, after which cell viability was examined using the CCK-8 assay. Values indicate mean ± SEM for at least three independent experiments performed in triplicate. **P* < 0.05, ***P* < 0.01, and ****P* < 0.001, ns = not significant.

**Supplemental Figure S2.The regimen combining chidamide and MI-3 is not effective in non MLL-r AML cells.** Human non MLL-rearranged AML cell lines KASUMI (**A**) and KG1a cells (**B**) were exposed to the indicated concentrations (μM) of chidamide and MI-3 alone or in combination for 48 hrs, after which the percentage of apoptosis was determined by flow cytometry. (**C**) KASUMI and KG1a cells were exposed to the indicated concentrations (μM) of and MI-3 alone or in combination for 24-72 hrs, after which cell viability was examined using the CCK-8 assay. Values indicate mean ± SEM for at least three independent experiments performed in triplicate.

**Supplemental Figure S3. Co-treatment with chidamide and MI-3 induces robust apoptosis, ROS generation, and loss of mitochondrial membrane potential in MLL-r MV4-11 cells. (A)** Human MLL-rearranged MV4-11 cells were treated with 2.5 μM chidamide ± 15 μM MI-3 for 48 hrs, after which the percentage of apoptotic cells was performed to determine by flow cytometry following Annexin V and PI double staining. **(B**, **C)** MV4-11 cells were treated as above, followed by the colony formation assay (**B**) and flow cytometric analysis of mitochondrial membrane potential (**C**). For panels A-C, the representative results were presented (left), and the data for at least three independent experiments was shown as bar graphs (right). **(D)** Alternatively, intracellular ROS levels were measured by flow cytometry using the Reactive Oxygen Species Assay Kit.. **(E)** Human non MLL-r KASUMI cells were treated with 5.5 μM chidamide ± 60.4 μM MI-3 for 48 hrs, followed by the colony formation assay. Values indicate mean ± SEM for at least three independent experiments performed in triplicate (****P* < 0.001).

**Supplemental Figure S4. The KEGG analysis of the RNAseq data reveals involvement of multiple cell cycle and DNA repair pathways in the interaction between chidamide and MI-3 in MLL-r AML cells.** MOLM-13 cells were treated with 2.63 μM chidamide ± 13.88 μM MI-3 for 48 hrs, after which total RNA was extracted and subjected to whole exome RNAseq. **(A-C)** The GESA analysis reveals the pathways the most significantly altered in MOLM-13 cells exposed to MI-3 (**A**) and 2.63 μM chidamide (**B**) alone or in combination (**C**). (**D**) The GO analysis was performed to reveal the most enriched GO terms of the transcripts up-regulated by chidamide with or without MI-3 but down-regulated by MI-3 as shown in **Figure 3E.** **(E)** The KEGG analysis was carried out to reveal the annotations of the most enriched pathways of the transcripts up-regulated by chidamide with or without MI-3 but down-regulated by MI-3 as shown in **Figure 3E**.

**Supplemental Figure S5. Genome-wide RNA sequencing identifies four genes that are differentially expressed in MOLM-13 cells treated with MI-3 vs. chidamide alone or in combination.** MOLM-13 cells were treated with 2.63 μM chidamide ± 13.88 μM MI-3 for 48 hrs, after which whole exome RNAseq was performed as described in Figure 3. The Dr. TOM analysis, an in-house customized data mining system of the BGI, was performed to identify genes that were down-regulated ≥2 folds by MI-3 or chidamide and ≥4 folds by combined treatment.

**Supplemental Figure S6. Treatment with chidamide results in increased acetylation of histone H3 in both MLL-r and non MLL-r AML cells. (A, B)** Human non MLL-r KASUMI cells (**A**) and MLL-r MV4-11 cells (**B**) cells were exposed to chidamide ± MI-3 for 48 hrs, after which Western blot analysis was performed to monitor the levels of total and acetylated histone H3.

**Supplemental Figure S1**

**Supplemental Figure S2**

**Supplemental Figure S3**

**Supplemental Figure S4**

**Supplemental Figure S5**

**Supplemental Figure S6**
